# Transcriptome Ortholog Alignment Sequence Tools (TOAST) for phylogenomic dataset assembly

**DOI:** 10.1186/s12862-020-01603-w

**Published:** 2020-03-30

**Authors:** Dustin J. Wcisel, J. Thomas Howard, Jeffrey A. Yoder, Alex Dornburg

**Affiliations:** 1 Department of Molecular Biomedical Sciences, NC State University, Raleigh, NC USA; 2 Comparative Medicine Institute, NC State University, Raleigh, NC USA; 3 Center for Human Health and the Environment, NC State University, Raleigh, NC USA

**Keywords:** BUSCO ortholog assembly, Cetacean and teleost fish phylogeny, Missing data visualization, Transcriptome, Concatenated alignment

## Abstract

**Background:**

Advances in next-generation sequencing technologies have reduced the cost of whole transcriptome analyses, allowing characterization of non-model species at unprecedented levels. The rapid pace of transcriptomic sequencing has driven the public accumulation of a wealth of data for phylogenomic analyses, however lack of tools aimed towards phylogeneticists to efficiently identify orthologous sequences currently hinders effective harnessing of this resource.

**Results:**

We introduce TOAST, an open source R software package that can utilize the ortholog searches based on the software Benchmarking Universal Single-Copy Orthologs (BUSCO) to assemble multiple sequence alignments of orthologous loci from transcriptomes for any group of organisms. By streamlining search, query, and alignment, TOAST automates the generation of locus and concatenated alignments, and also presents a series of outputs from which users can not only explore missing data patterns across their alignments, but also reassemble alignments based on user-defined acceptable missing data levels for a given research question.

**Conclusions:**

TOAST provides a comprehensive set of tools for assembly of sequence alignments of orthologs for comparative transcriptomic and phylogenomic studies. This software empowers easy assembly of public and novel sequences for any target database of candidate orthologs, and fills a critically needed niche for tools that enable quantification and testing of the impact of missing data. As open-source software, TOAST is fully customizable for integration into existing or novel custom informatic pipelines for phylogenomic inference.

Software, a detailed manual, and example data files are available through github carolinafishes.github.io

## Background

Advances in sequencing technology have dramatically decreased the cost of transcriptomic sequencing, driving a rapid increase in the representation of non-model organisms in transcriptome public databases. This proliferation of sequence data has generated tremendous opportunities for studies that span the molecular evolution of gene families [1, 2] to human disease [3, 4]. Nevertheless, publicly available transcriptomic databases remain underutilized in phylogenomic applications. This is unfortunate, as orthologous markers assembled from public transcriptome data have been shown to provide a cost-effective means to resolve some of the most vexing problems in the Tree of Life [5–7]. A major impediment for using public transcriptomic data in phylogenomics has been the lack of ease in implementing bioinformatic tools for ortholog identification. However, software for Benchmarking Universal Single-Copy Orthologs (BUSCO) [8] provides a powerful framework from which to develop much needed tools for aggregating these orthologs for phylogenomic studies.

BUSCO was originally designed to estimate the “completeness” of genome sequences and whole transcriptome datasets by assessing the number of orthologs expected to be present in all species belonging to a selected clade from a list found in the OrthoDB [8]. Performing BUSCO analysis on multiple species results in the annotation of transcripts or genes with universal identifications (IDs) that could be used as a basis for aggregating sequences for later use in phylogenomic analyses [9]. However, the absence of easy to use, peer reviewed software tools targeted towards the phylogenetics community prohibit the widespread adaptation of this approach. In addition to the original python scripts used to harvest BUSCO orthologs for phylogenetic studies [9], we are aware of only two other well-documented pipelines that exist on private websites or github. The first pipeline, QKbusco [10], is a set of python scripts that the user calls sequentially. The second is part of an on-line bioinformatics tutorial that relies heavily on bash loops and user input of command line prompts such as “sed” and “awk” [11]. While these three pipelines are easy to access, they require a high level of input and computational experience from the user across multiple sequence ‘search & query’ and ‘assembly’ steps. Even for experienced users, such a process is not efficient. Additionally, the resulting sequences still require significant amounts of processing prior to tree inference. Clearly needed is a software package that automates the fetching and alignment of BUSCO-identified orthologs from transcriptomic data in order to empower the community of evolutionary biologists to effectively harness the potential of this growing set of sequence data.

Here we present ‘Transcriptome Ortholog Alignment Sequence Tools (TOAST)’, a versatile and efficient R package for aggregating single-copy orthologs from either public and/or local transcriptomic resources of targeted organisms and aligning those sequences for subsequent phylogenomic studies. For a given clade, nucleotide sequences from the NCBI database [12] or within a directory on the user’s hard drive can be accessed and assigned a universal ortholog ID from OrthoDB using BUSCO [13]. From this annotated data, we can orient (e.g. 5′ to 3′ direction of transcripts) and align sequences to each other using existing alignment methods [14]. Using returned alignments, TOAST facilitates visualization of missing data patterns, and options are available for generating additional alignments based on user specified levels of data matrix completeness, including customizable concatenated datasets with a corresponding partition file that can be fed directly into the phylogenetic software IQtree [15] for partition and model selection as well as subsequent tree inference.

## Implementation

TOAST was designed to be run locally, e.g., on a laptop or desktop with modest capabilities. Most of TOAST’s functionality (data fetching and alignment) is currently designed for UNIX systems to accommodate the UNIX reliancy of BUSCO and it’s dependencies (Linux/Mac) [16]. As BUSCO utilizes parallel processing, advanced users may speed up the BUSCO step by utilizing a computer cluster to perform this analysis across more cores and then moving the result files to a local machine. TOAST begins by downloading cDNA sequence fasta files from species within a specified taxonomy group. These fasta files include everything in the National Center for Biotechnology Information (NCBI) nucleotide database which includes all transcriptome shotgun assembly sequence database (TSA) sequences for each species. These sequences are stored in taxonomically informed file name, *genus_species*.fasta, within the designated folder. Using internal functions, TOAST employs BUSCO v3.0.2 [9, 13], along with HMMER v3.1b2 [17] and NCBI BLAST+ [18] to find orthologs within the selected OrthoDB database [9, 13]. TOAST will input all of the fasta files from the specified directory, run each through BUSCO, and write the results in a new directory. TOAST next parses the information from the full table of ortholog matches within the results folder for each organism. Both complete and fragmented BUSCO IDs are retained and users are able to specify an acceptable degree of fragmentation to retain based on the expected length of target loci. In the case of duplicated results, the best scoring sequence is reported. In the event duplicated sequences have the same score, the first sequence encountered is reported. Sequences from each organism are binned into fasta files based on BUSCO IDs. It is important to note that TOAST is not limited to only analyzing public data as TOAST can also be used to fetch and align orthologs from local transcriptomes or combine local searches with online fetching. While we use public data for illustrative purposes here, we envision the primary utility of this option will be to empower streamlined integration of public and novel sequence data.

The reported fasta files contain the best BUSCO nucleotide sequence for each organism. However, the direction of the DNA sequence may be reversed for some species. Therefore, TOAST uses MAFFT [14] to both align the sequences, and assure that all sequences are oriented in the same direction. Individual alignment files are written to relaxed phylip format, and are ready for use with phylogenetic software such as IQ-TREE [15]. These alignments can be concatenated into a single alignment using the TOAST function “SuperAlign”. In addition, the location of sequence partitions within this supermatrix can be written using the TOAST function “PartitionTable” in the nexus format read by IQ-TREE [15].

As given loci will vary with regard to their representation across target taxa, TOAST users have a series of options from which to visualize missing data patterns as well as the distribution of fragmented loci. These include missing data patterns across all loci, as well as missing data patterns for user defined hierarchical levels (i.e., taxonomy). These functions work both in UNIX environments as well as windows. TOAST additionally has the ability to compute missing data across a directory of fasta files for any set of loci (e.g., anchored hybrid enrichment [19–21], ultraconserved elements [22–24] etc), filling a critical software need for phylogeneticists. Based on the user defined criterion of acceptable levels of missing information, TOAST can omit taxa from the original alignment, realign each locus, and provide a new concatenated matrix and associated partition block file that defines the location of each locus. These files can be directly read into IQ-TREE [15] for inference.

## Results & discussion

We demonstrate the utility of our program and also explore potential limitations and pitfalls of BUSCO orthologs for phylogenetics by analyzing three datasets that illustrate different classes of phylogenetic problems. First we test the utility of TOAST for resolving the earliest divergences within delphinoids, a clade that experienced a geologically recent pulse of diversification [25–27]. Second, we test whether strongly supported resolution of the sister lineage to teleost fishes is achievable using BUSCO orthologs. This dataset spans an evolutionary timescale on par with some of the earliest divergences within major groups of living jawed vertebrates. Finally, we assess the effect of tissue specific expression patterns on the representation of BUSCO orthologs between two closely related species of camelids. This third dataset provides expectations of data coverage heterogeneity and also demonstrates the ability of TOAST to link recovered loci to the gene ontology (GO) database to assess how the estimated functional roles of loci impact expression patterns.

### Resolving the early divergences of delphids

**Fig. 1:**
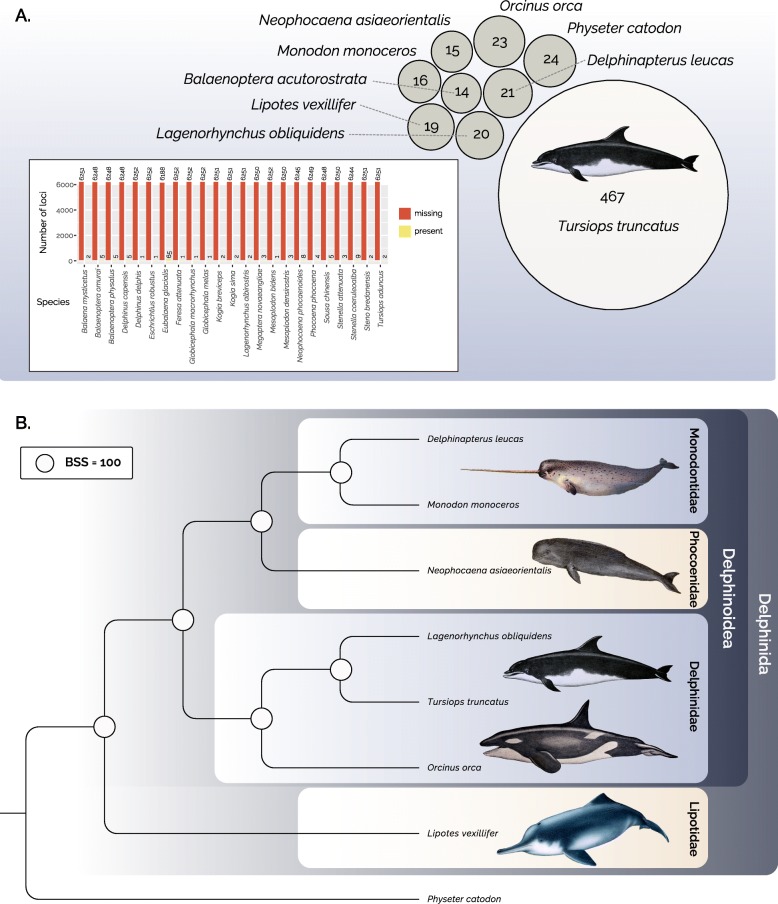
Visualization of missing data patterns and estimation of delphinid phylogeny enabled by TOAST. **a** Circle pack plot showing the number of missing loci within taxa that contain at least 1000 loci, contrasted with a barplot of missing (red) versus present (yellow) data patterns within taxa that do not contain at least 1000 loci (insert). **b** Maximum likelihood phylogeny of delphinid lineages inferred from TOAST harvested BUSCO loci using IQTree. Circles at nodes represent bootstrap support (BSS) values of 100. Delphid images modified from public domain illustrations

We accessed nucleotide and TSA sequences for each species of Cetacea (whales and dolphins; NCBI ID = 9721) and using the laurasiatheria_odb9 dataset (database of single-copy laurasiatherian orthologs that are present in at least 90% of species) from the OrthoDB website [9, 13]. Using TOAST, we create a set of files for each species that includes gene IDs that match specific BUSCO IDs from the laurasiatheria_odb9 dataset. The representation of ortholog identification within a species varied from complete coverage (6253 loci) to less than 14 loci. However, exploration of missing data patterns demonstrates higher levels of missing data within toothed whales. Using an arbitrary threshold of including only cetaceans with at least 1000 of the over 6000 possible orthologs revealed that most missing data was localized in the dolphin *Tursiops* (Fig. 1a), and that most taxa not meeting this threshold contained very few loci (Fig. 1a). Further visualizations possible with TOAST demonstrate that this threshold of minimally containing 1000 loci would remove the majority of the missing data the concatenated alignment (Fig. 1b, Supplemental materials).

Using this threshold, we constructed a concatenated alignment of 1,473,632 nucleotides for representatives of the major delphinid lineages Monodontidae (*Delphinapterus leucas* and *Monodon monoceros*), Phocoenidae (*Neophocaena asiaeorientalis*), Delphinidae (*Lagenorhynchus obliquidens*, *Tursiops truncatus*, *Orcinus orca*), and Lipotidae (*Lipotes vexillifer*) using *Physeter catodon* as an outgroup. Maximum likelihood based phylogenetic tree searches were conducted using this data in conjunction with best fit nucleotide substitution models and partitions chosen using Bayesian Information Criteria (BIC) in IQ-TREE [15]. Confidence in the topological inference was assessed using 1000 bootstrap replicates (Fig. 1b). This resulting tree provides strongly supported topological resolution for the evolutionary relationships of major delphinid lineages, supporting previous hypotheses of a sister group relationship between Delphinidae and a clade comprised of Monodontidae + Phocoenidae [28–30] Our analyses also strongly support an early divergence of *Lipotes* prior to the radiation of delphioids (Fig. 1b). This result is consistent with previous phylogenetic analyses that suggested *Lipotes* to be one of several deeply divergent lineages of river dolphins that invaded freshwater in the Miocene [31–33]. During this period, sea level rise led to the creation of shallow seaways that provided new estuarine habitats for cetaceans on several continents. Subsequent lowering sea levels is thought to have isolated these lineages from their marine relatives, resulting in the independent evolution of modern river dolphins [32]. Our phylogenetic results are consistent with other topological inferences that have been used as a basis to hypothesize that river dolphins such as *Lipotes* are vestiges of previously diverse marine cetacean clades that were replaced by the radiation of delphinoids [32], and simultaneously demonstrate the potential utility of TOAST for generating phylogenetic datasets for recent radiations.

### Resolving the sister lineage to teleosts

**Fig. 2:**
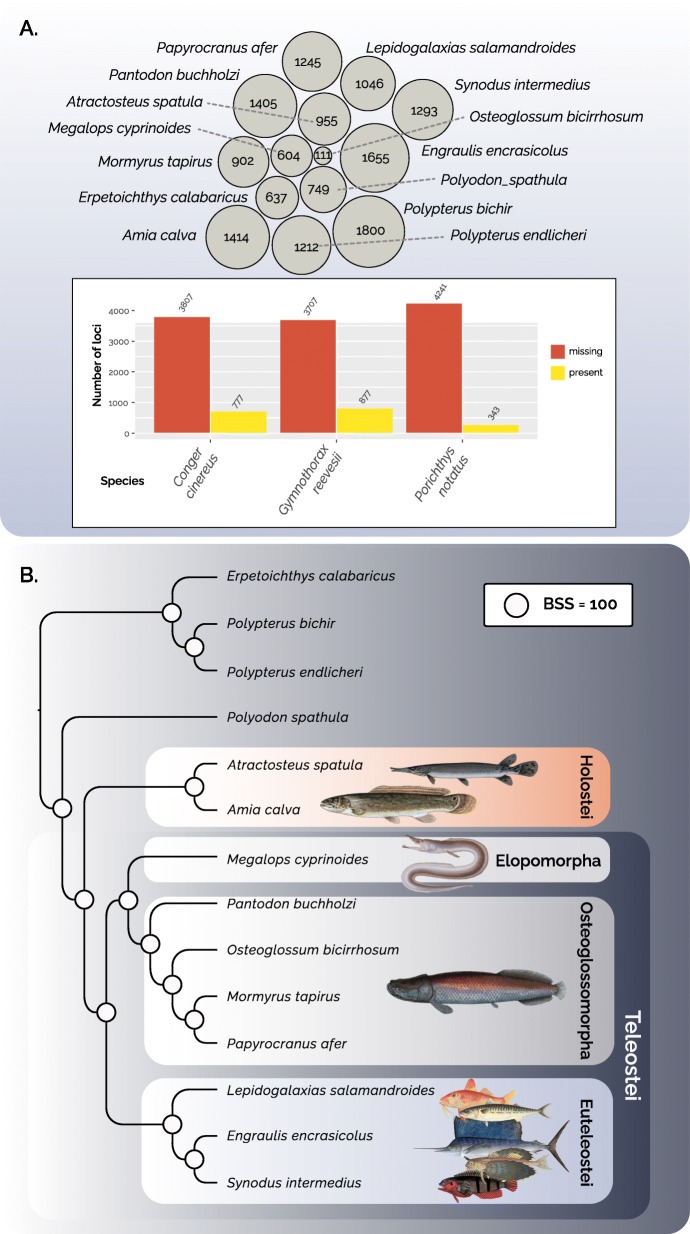
Visualization of missing data patterns and estimation of early ray-finned fish phylogeny enabled by TOAST. **a** Circle pack plot showing the number of missing loci within taxa that contain at least 1000 loci, contrasted with a barplot of missing (red) versus present (yellow) data patterns within taxa that do not contain at least 1000 loci (insert). **b** Maximum likelihood phylogeny of ray-finned fish lineages inferred from TOAST harvested BUSCO loci using IQTree. Circles at nodes represent bootstrap support (BSS) values of 100. Fish images modified from public domain illustrations

We used transcriptomes of representative species of ray finned fishes from a recent study by Hughes et al. [34] to resolve the sister lineage to teleost fishes. Species selected span all major clades of non-teleost fishes, including representative bichirs (*Erpetoichthys calabaricus*, *Polypterus bichir*, *Polypterus endlicheri*), paddlefish (*Polyodon spathula*), gar (*Atractosteus spatula*), and bowfin (*Amia calva*). Teleosts were selected to include major divergences in the earliest diverging teleost lineages elopomorpha (*Megalops cyprinoides*, *Gymnothorax reevesii, Conger cinereus*) and osteoglossomorpha (*Osteoglossum bicirrhosum, Mormyrus tapirus, Pantodon buchholzi, Papyrocranus afer*), as well as several euteleost species (*Engraulis encrasicolus, Lepidogalaxias salamandroides, Synodus intermedius, Porichthys notatus*). Using the Actinopterygii_odb9 dataset (database of single-copy 4584 orthologs that are present in at least 90% of species) from the OrthoDB website [9, 13], we then used Using TOAST to harvest gene IDs that had a match to specific BUSCO IDs (4529 loci). Quantification of missing data patterns revealed that almost taxa had greater than 70% representation. However, high levels of missing data was localized to three species (*Gymnothorax reevesii*: 81% missing, *Conger cinereus*: 84% missing, and *Porichthys notatus*: 94% missing Fig. 2a). We constructed a concatenated alignment of 24,225,167 nucleotides for all taxa excluding these three species with high missing data values. We conducted a maximum likelihood analyses using best fit models and partitions selected with BIC in IQ-TREE with 1000 bootstrap replicates (Fig. 2b). The resulting tree provides strongly supported topological resolution for every node, supporting previous hypotheses of a sister group relationship between teleosts and Holostei, a clade comprised of gars and bowfin.

For over 150 years, gars and bowfin have been repeatedly hypothesized to form a natural group: Holostei. Originally defined to include a combination of bichirs, gar, and bowfin [35], a revision by Huxley [36] redefined the clade to comprise only bowfin and gar. However, anatomical investigations in the twentieth century have repeatedly challenged the validity of Holostei, often proposing instead that bowfin share a closer affinity to teleosts than gar, though recent work by Grande [37] again revived Holostei as sister to teleosts. In sharp contrast to decades of debate among morphologists [38–41], molecular phylogenetic analyses have been nearly unanimous in resolving a strongly supported holostei in studies using mtDNA genomes [42–44], nuclear exons [42, 45–47], as well as transcriptomic and genomic analyses [34, 48]. Such congruence is surprising across years of independent study, and our results add yet another line of evidence strongly supporting the monophyly of Holostei (Fig. 2).

The resolution of Holostei as sister to teleosts has important implications for biomedical research using teleost model organisms. Linking genomic work in models such as zebrafish, sticklebacks, or medaka back to humans is often challenged by factors that include the loss of ohnologs following the early rounds of vertebrate genome duplication [49, 50], duplicates of loci found in humans as a consequence of the teleost genome duplication [51], and generally rapid rates of molecular evolution in target loci of many teleosts [52, 53]. However, sequencing of the genome of spotted gar (*Lepisosteus oculatus*) has revealed surprising similarity between this holostean genome and genomic features of both teleosts and tetrapods [48]. For example, analysis of the major histocompatibility (MHC) genes in gar revealed synteny to human loci as well as identification of other major groups of immune receptors thought to be teleost specific [48]. Similarly, the gar genome contains sequence information that can link hidden orthology of teleost and human microRNAs as well as conserved non-coding elements (CNEs) that appear to be highly informative for understanding the “fin to limb” transition [48]. Further investigation of gar CNEs also linked numerous human disease-associated haplotypes to locations within the zebrafish genome, providing new opportunities for functional experiments [48]. The biomedical importance of the genome of a single gar in combination with the recognition of holostean monophyly raises the question of what discoveries await discovery in not only the genomes of more gar, but also the bowfin genome. Given that both the fossil record and molecular clock studies point to an ancient divergence of gar and bowfin several hundred million years ago [45, 46], investigation of the bowfin genome is sure to illuminate not only biomedically relevant research, but also fundamental aspects of vertebrate genome biology.

### Assessing tissue-level expression data in camelids

Transcriptome sequences were downloaded from NCBI’s TSA for two camel species (Cambac:*Camelus bactrianus* and Camdro:*Camelus dromedarius*) and eight matching tissues (brain: GAES01|GADT01, heart: GAET01|GADU01, hypothalamus: GAEU01|GADV01, kidney: GAEV01|GADW01, liver: GAEW01|GADX01, lung: GAEX01|GADY01, muscle: GAEY01|GADZ01, skin: GAFA01|GAEA01, and testis: GAEZ01|GAEB01). Utilizing these transcriptome datasets, we demonstrate TOAST’s ability to compare patterns data coverage within the same species and between species and/or tissue types and compare these patterns to expectations of predicted function.

**Fig. 3:**
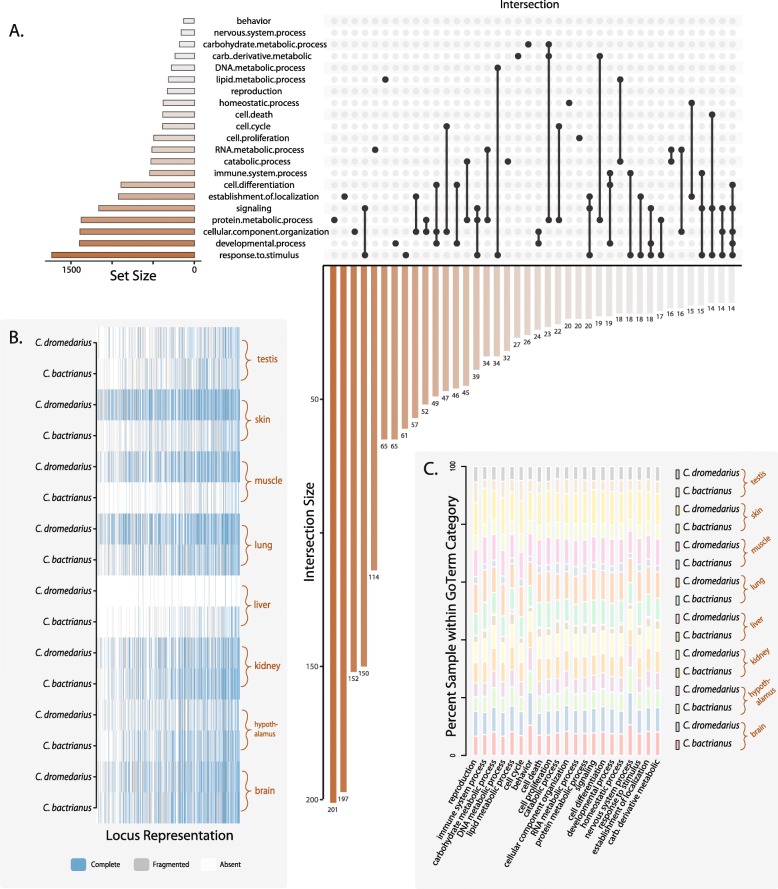
Visualization of the representation and intersection of GoTerms between tissues of *Camelus dromedarius* and *C. bactianus* using TOAST. **a** Upset plot showing the intersection of GoTerms across all loci. Set size represents the number of loci found within each specific category. Intersection size (lower histogram) represents the number of loci within each intersection column. **b** Occupancy matrix of complete (blue), fragmented (gray), and missing (white) loci between species and specific tissues. **c** Relative percent of GoTerms sampled between tissues and species

TOAST builds upon the R packages GSEABase and GOstats [54] to assign the orthologs within a given BUSCO database into Gene Ontology Terms (GoTerm) categories. For the camelids, we used TOAST to organize the Laurasiatheria_odb9 database [55, 56], and display GoTerm assignation and overlap (GoTerms) using the dependent R package UpSetR [57] (Fig. 3a). Our results demonstrate that the BUSCO loci for this subset of mammals are largely involved in developmental processes and signalling responses (Fig. 3a). We demonstrate different degrees of missing data between species and tissue samples, with generally high numbers of fragmented loci in the testis and and very little coverage of loci within liver samples of both species (Fig. 3b). Additionally, there appear to be minor differences in the relative percent of GoTerms sampled in brain tissues with the GoTerm “Development” to compared to other tissue types (Fig. 3c). Depending on user goals, TOAST offers users the ability to remove fragmented loci based on a length threshold. In this case, our results demonstrate the heterogeneity of BUSCO coverage that can occur between the same tissue types of closely related species. Although it is premature to interpret biological significance from missing data patterns of this dataset, our results demonstrate the potential for BUSCO coverage limitations stemming from tissue and lineage specific patterns of expression.

## Conclusions

TOAST provides a comprehensive set of tools for assembly of sequence alignments of orthologs from public and/or local transcriptomic datasets, enabling streamlined assembly of sequence datasets for any target database of candidate orthologs. TOAST not only allows for effective capture of public data, but can also be used to integrate novel sequencing with existing (public or private) transcriptomic data. As BUSCO datasets now capturing lineages that span vertebrates to arthropods, fungi, and bacteria, TOAST offers a versatile framework for incorporating transcriptomic resources and BUSCO orthologs into phylogenetic studies. Currently, TOAST is designed for working with transcriptomic data. However, intrepid users could also gather predicted genes from genomic assemblies for compiling BUSCO orthologs in TOAST. Our results reveal the potential utility of these loci for phylogenetic problems of spanning different temporal scales. However, we also demonstrate the potential for heterogeneous sequence coverage between species and tissue types necessitating assessment of missing data patterns.

Output from TOAST facilitates visual and quantitative assessment of missing data patterns that can be integrated with existing approaches to quantify matrix decisiveness [58] or phylogenetic information content [59]. Missing data visualization functions are designed to work with any delimited file of data presence/absence such as behavioural, phenotypic, gene expression, etc. studies. As missing elements within a matrix are a common feature of large datasets, TOAST provides a useful set of tools for visually scrutinizing data and considering the potential for biases, such as tree terracing in phylogenetic inference [58]. Using interactive TOAST functions, users can determine acceptable thresholds for minimum representation in the sequence data matrix and readily subsample their data along preset criteria. As such, TOAST empowers phylogeneticists to effectively harness the potential of transcriptomic data as well as investigate the impact of missing data patterns on inferences, filling two important niches of high utility for resolution of a genomic Tree of Life.

## Availability and requirements

Lists the following:

Project name: TOAST

Project home page: https://github.com/carolinafishes/TOAST

Operating system(s): UNIX (Mac and Linux)

Programming language: R

Other requirements: R 3.6 or higher, Python 3 or higher, BUSCO, HMMer, BLAST, and Mafft installed

License: e.g. GNU GPL 3

Any restrictions to use by non-academics: license needed

## Supplementary information

**Additional file 1 .**

## Data Availability

Software with an additional manual and example data used in the text is hosted on github: https://github.com/carolinafishes/TOAST and an online tutorial is hosted here https://carolinafishes.github.io/software/TOAST/

## Abbreviations

TOAST: Transcriptome Ortholog Alignment Sequence Tools
BUSCO: Benchmarking Universal Single-Copy Orthologs
IDs: Identifications
TSA: Transcriptome shotgun assembly sequence database
NCBI: National Center for Biotechnology Information
BIC: Bayesian Information Criterion
