# Supplementary material for: Transcriptome Ortholog Alignment Sequence Tools (TOAST) for phylogenomic dataset assembly
Source: BMC Evol Biol. 2020 Mar 30;20:41. doi: 10.1186/s12862-020-01603-w (PMC7106827; doi:10.1186/s12862-020-01603-w)
Supplement: Supplementary file 1 — Additional file 1 . [file 12862_2020_1603_MOESM1_ESM.pdf]

Transcriptome Ortholog

T·O·A·S·T

Alignment Sequence Tools

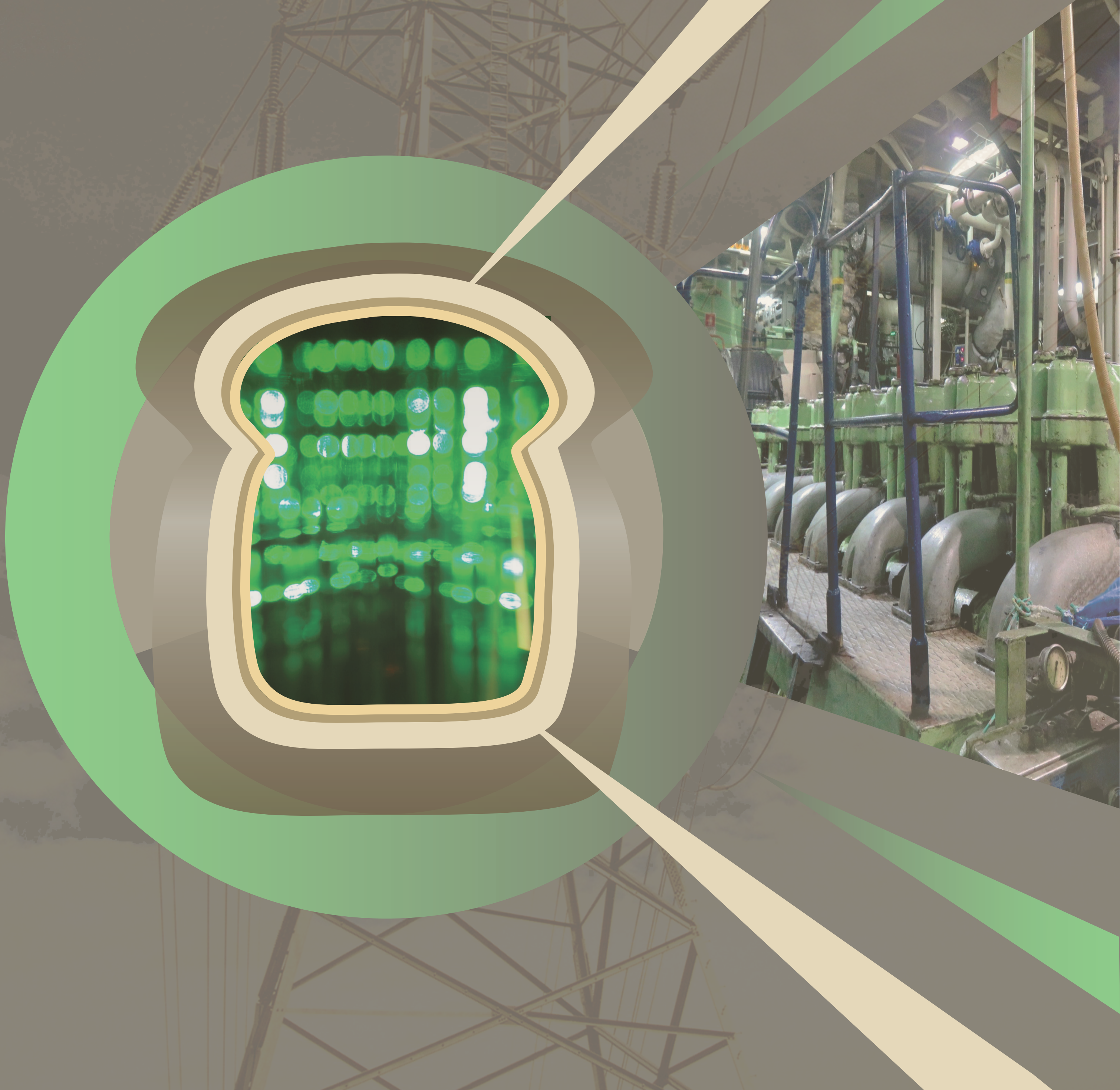

Wcisel • Howard • Yoder • Dornburg

Updates to TOAST and the manual are available at
carolinafishes.github.io/software/TOAST/

Please check your release version to make sure you are not missing out on new features

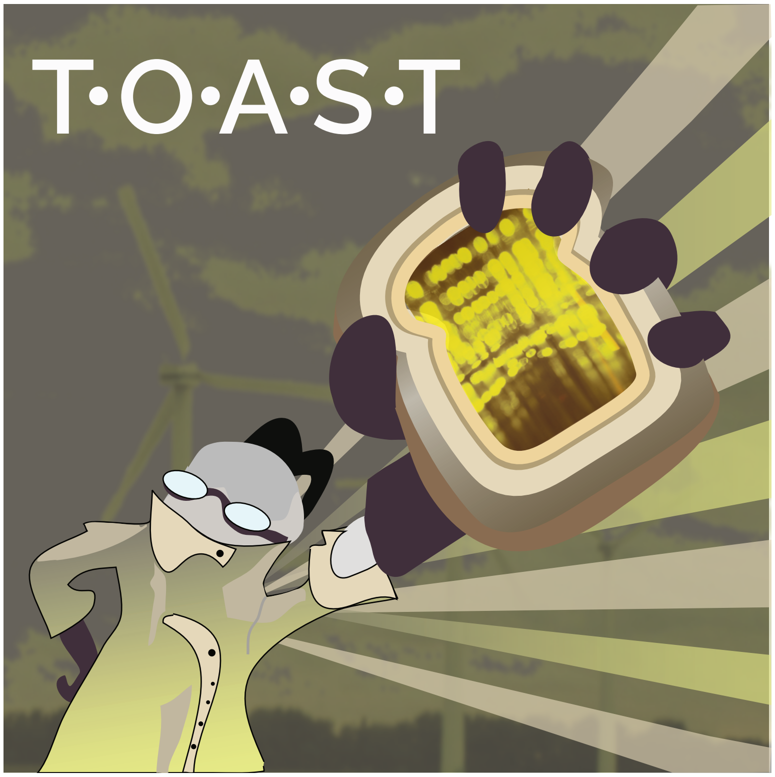

Copyright 2019 Dustin Wcisel, Thomas Howard, Jeffrey A. Yoder, and Alex Dornburg

This document is part of TOAST

TOAST is free software: you can redistribute it and/or modify it under the terms of the GNU General Public License as published by the Free Software Foundation, either version 3 of the License, or (at your option) any later version.

TOAST is distributed in the hope that it will be useful, but WITHOUT ANY WARRANTY; without even the implied warranty of MERCHANTABILITY or FITNESS FOR A PARTICULAR PURPOSE. See the GNU General Public License for more details.

You should have received a copy of the GNU General Public License along with TOAST.
If not, see <http://www.gnu.org/licenses/>.

Images: Copyright 2019 Alex Dornburg

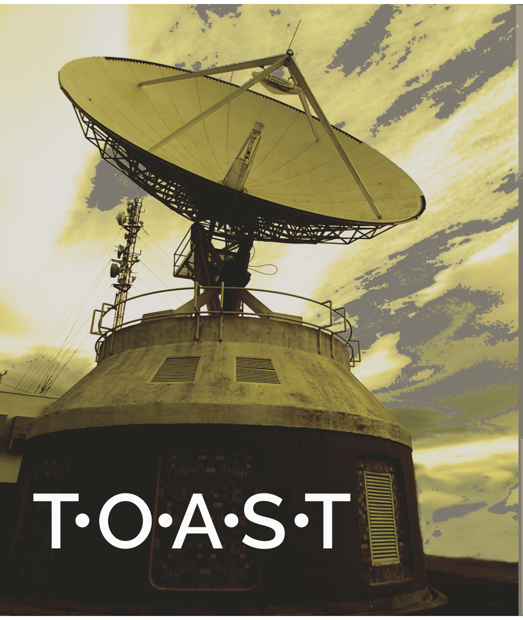

Tools for Harnessing Next-Generation Sequence Data

D. Wcisel, T. Howard, J. Yoder, and A. Dornburg

Table of Contents

Section 1

Installation 4

Overview 7

Section 2

Harvesting online orthologs 8

Section 3

Harvesting orthologs from local fasta files 10

Merging novel data with public data 10

Section 4

Visualizing missing data patterns ... ... .11

Assembly of sequence datasets ... ... .17

Section 5

Advanced Visualizations ... ... .18

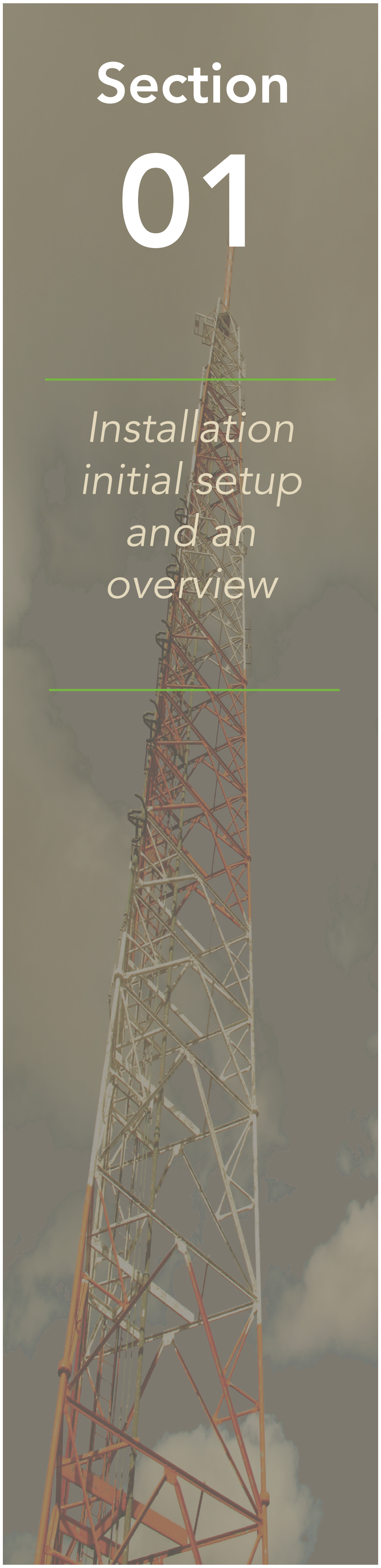

Installation and setup

TOAST is hosted on Github as an R package with additional links and documentation found at the following url
carolinafishes.io/software/toast

Toast utilizes two R packages currently not available on CRAN. Installing these dependencies prior to loading the TOAST for the first time is required. Fortunately, TOAST can by installed using devtools which automates this process

WARNING

TOAST requires R version 3.6.1 or higher for installation of dependencies and functionality. Update R prior to going any further

Once you have made sure to have an appropriate version of R you can install TOAST as follows

```
#in case you need devtools
install.packages("devtools")

library(devtools)
devtools::install_github("carolinafishes/toast")
library("toast")
```

R Dependencies Overview

TOAST would not be possible without the efforts of other developers. The above code should automatically install all R based dependencies that you do not already have installed during the initial setup.

The primary packages that supply the foundation for TOAST in R are: rentrez[1] and gplots[2]

LINUX USERS ONLY: Please note that rentrez makes use of several dependencies that may not be on your computer. If you encounter errors during the installation of this package here is a list of dependencies you may need to install as follows in terminal

```
sudo apt install openssl
sudo apt install libssl-dev
sudo apt install libcurl4-opensll-dev
sudo apt install libxml2-dev
```

For visualizing patterns of missing data TOAST will also install the R packages ggplot2[3], devtools[4], viridis[5], packcircles[6], gridExtra[7],

data.tree[8], circlepackeR[9], foreach[10], doParallel[10], seqinr[11], reshape[12], usethis[13], and streamgraph[14] .

Additional Dependencies for Unix Users

BUSCO[15] is limited to only running on Unix (Mac/Linux) machines. If you plan to use TOAST to visualize missing data patterns, these functions will work on any platform (including Windows).
However , you need to be using Linux or Mac to use any functions that involve BUSCO harvesting of data (Next two sections of the manual). Further, MafftOrientAlign requires a Unix (Linux or OSX) environment.

TOAST requires you install MAFFT[16]
and place this into your global path (e.g., modify your ~/.bashrc file)

TOAST also makes use of BUSCO[15] , HMMER[17] and BLAST

Installing all of these prior to running is required!

Please see the BUSCO manual for instructions on installation. There is also a handy quickstart guide to installation at carolinafishes.github.io/miscr/busco4mac

Note that BUSCO recommends specific releases of HMMER and BLAST. But, we have found more recent versions of HMMER to work while the lastest BLAST (2.9+) will occassionally crash.
We recommend following the BUSCO instructions to ensure compatibility. There are several options available when installing BUSCO and we suggest using the following command to ensure no mismatch of python versions in R (This is particularly important for mac users!)

```
sudo python3 setup.py install
```

Reminder: Specific Modification of BUSCO

After you have successfully installed BUSCO you will need to copy and modify the config.ini file to direct BUSCO to both BLAST and HMMER.
Please see the BUSCO manual and follow these directions carefully.

CAUTION

CHECK that you have successfully installed BUSCO
prior to proceeding any further!

Section 01

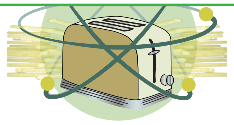

References

1. Winter, D.J., 2017. rentrez: An R package for the NCBI eUtils API (No. e3179v1). PeerJ Preprints.

2. Warnes, M.G.R., Bolker, B., Bonebakker, L. and Gentleman, R., 2016. Package ‘gplots’. Various R Programming Tools for Plotting Data.

3. H. Wickham. 2016. ggplot2: Elegant Graphics for Data Analysis. Springer-Verlag New York.

4. H. Wickham and W. Chang. 2017. devtools: Tools to Make Developing R Packages Easier. R package version 1.13.3.

5. S.Garnier. 2017. viridis: Default Color Maps from 'matplotlib'. R package version 0.4.0.

6. M. Bedward, D. Eppstein and P. Menzel. 2018. packcircles: Circle Packing. R package version 0.3.3.

7. B. Auguie. 2016. gridExtra: Miscellaneous Functions for "Grid" Graphics. R package version 2.2.1.

8. C. Glur. 2018. data.tree: General Purpose Hierarchical Data Structure. R package version 0.7.8.

9. J. Froelich. 2015. circlepackeR: htmlwidget for Mike Bostock d3.js circle packing visualization. version 0.1.0.9

10. S. Weston and R. Calaway. 2015 Getting Started with doParallel and foreach. https://cran.r-project.org/web/packages/doParallel/vignettes/gettingstartedParallel.pdf

11. Charif, D. and Lobry, J.R., 2007. SeqinR 1.0-2: a contributed package to the R project for statistical computing devoted to biological sequences retrieval and analysis. In Structural approaches to sequence evolution (pp. 207-232). Springer, Berlin, Heidelberg.

12. H. Wickham, 2007. Reshaping data with the reshape package. Journal of statistical software, 21(12), pp.1-20.

13. Wickham, H., 2015. R packages: organize, test, document, and share your code. " O'Reilly Media, Inc.".

14.Rudis, B. 2019. Streamgraph R package. https://hrbrmstr.github.io/streamgraph/

15. Simão, F.A., Waterhouse, R.M., Ioannidis, P., Kriventseva, E.V. and Zdobnov, E.M., 2015. BUSCO: assessing genome assembly and annotation completeness with single-copy orthologs. Bioinformatics, 31(19), pp.3210-3212.

16. Katoh, K. and Standley, D.M., 2013. MAFFT multiple sequence alignment software version 7: improvements in performance and usability. Molecular biology and evolution, 30(4), pp.772-780.

17. Eddy SR. A new generation of homology search tools based on probabilistic inference, Genome Inform. , 2009, vol. 23 (pg. 205-211)

Section 01

Quick Overview - Suggested Project Structure

TOAST will move between directories on your harddrive generating files. We recommend the following folder architecture to keep your project organized

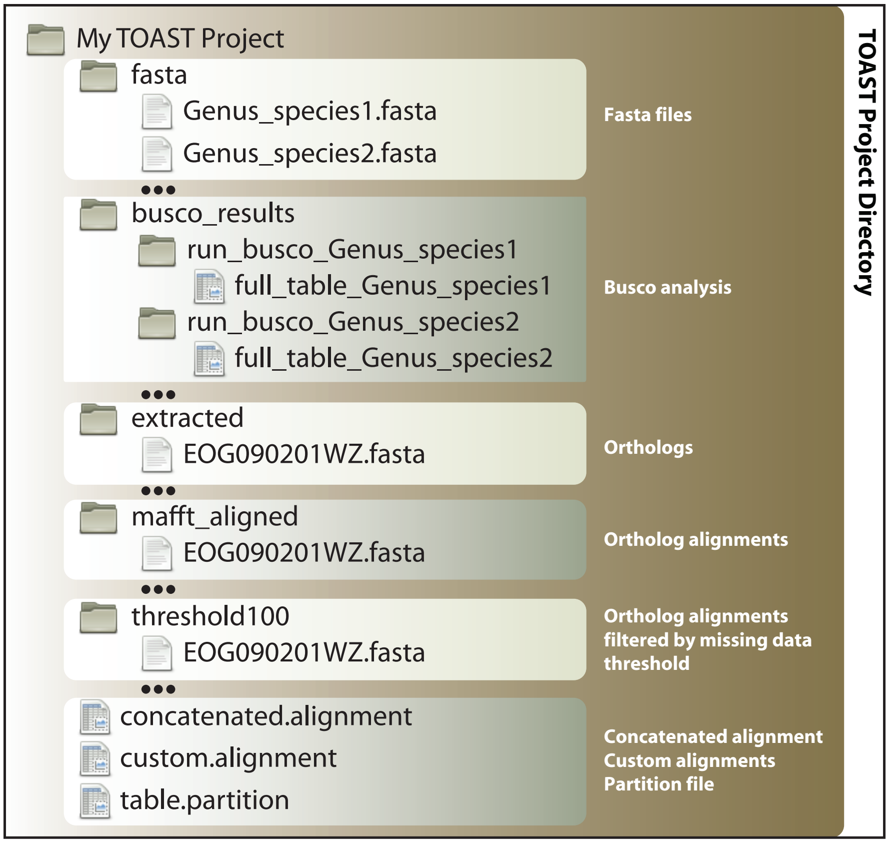

Within a main project folder,

1) Create a separate folder in which you store FASTA files. TOAST will also place downloaded FASTA files here.

2) Have a busco_results folder for storing and retrieving busco IDs

3) An extracted folder for your raw ortholog sequences

4) a mafft_aligned folder for your alignments of each ortholog

5) a threshold folder for you alignments of each ortholog with species removed due to not meeting requirements of representation

TOAST will write concatenated alignments, custom concatenated alignments of user selected loci, and partition files into the main directory for easy retrieval.

Function Overview

Here is an overview of core functions and their purpose

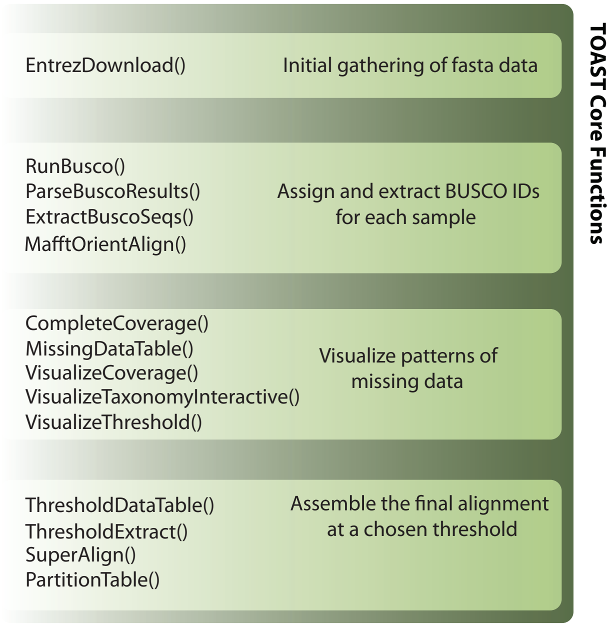

TOAST functions can be divided into 4 groups

1) Sequence gathering functions

2) BUSCO related functions

3) Missing data pattern visualization

4) Alignment assembly

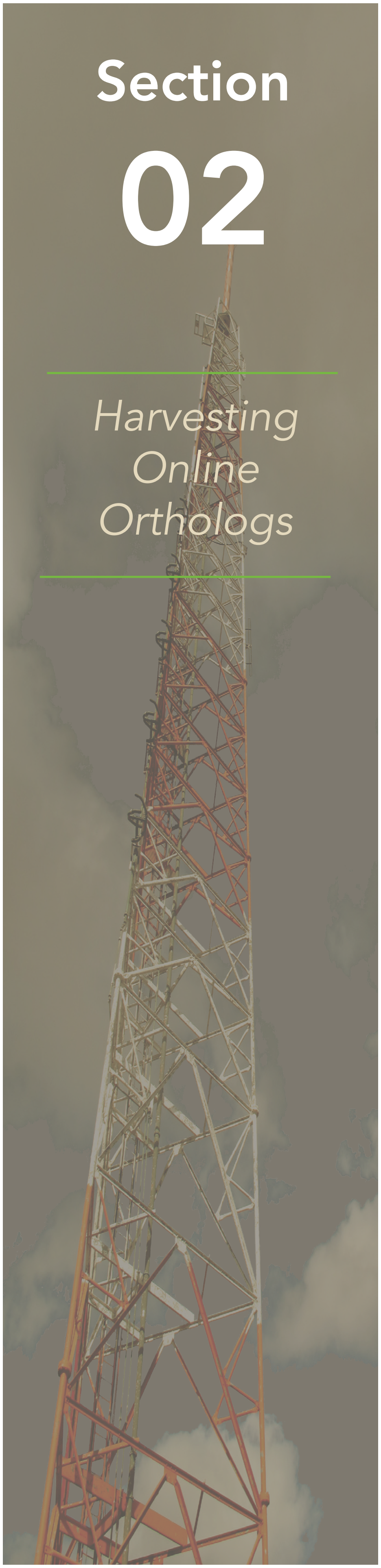

Harvesting New Ortholog Datasets

For a given focal clade (mammals, cetaceans, squirrels, etc), TOAST will utilize BUSCO and BLAST to download, find, and extract all orthologs from available public data on NCBI.

Use of this method requires a
Taxonomic ID for your focal clade

The taxonomic ID can be easily obtained from NCBI as follows
1) Navigate to https://www.ncbi.nlm.nih.gov/taxonomy/
2) In the Taxonomy search field enter your desired clade
3) Click on the result. This will give you a full breakdown of subclade taxonomy. Click on the link for the clade you want to focus on.
4) This will send you to a page that includes the taxonomic ID. In the case of Cetacea this is 9721.
Once you have this you are ready to start!

GETTING STARTED

To use BUSCO and harvest orthologs you need to be using LINUX and also have installed the dependencies mentioned in section 1. If you said yes to both of these things, then we are ready to begin.

Looking at the example_script.R file, you will see that you need to define some locations as well as the number of cores to use.

```
#universal variables
td <- "/home/dustin/temp/trial1" #toast_directory
fd <- "/home/dustin/temp/trial1/fasta" #fasta_dir
bs <- "/home/dustin/software/busco/scripts/run_BUSCO.py" #path to busco_script
bd <- "/home/dustin/temp/trial1/busco_results" #path to busco results directory
ed <- "/home/dustin/temp/trial1/extracted" #extracted_dir
md <- "/home/dustin/temp/trial1/mafft_aligned" #mafft_dir
od <- "/home/dustin/temp/trial1/350_laurasiatheria_odb9" #path to orthoDB directory
ad <- "/home/dustin/temp/trial1/mafft_aligned" #mafft_dir, which is a directory of aligned fastas
cpu <- 12 #number of threads to use at various steps
```

To explain, you need to provide the location of toast (td), the location of where to store and or find your FASTA files (fd), the location of BUSCO (bs), where to store your BUSCO results (bd), where to store extracted orthologs (ed), where to store alignments (md), where the ortholog database is (od), where to store/find aligned FASTA files. We recommend working on a directory per project as above (page 7), but leave that choice up to you.

Step 1 Download Data

Once you have set up your paths, you can download sequences as follows

```
EntrezDownload(txid = 9721, fasta_dir = fd, minimumSeq = 350, maximumSeq = NULL)
```

Using a taxonomy id and a specified fasta directory, this is will download all databases per species with at least X number of sequences specified by minimumSeq. By default minimumSeq is set to 350. In addition, if you wish to simply test that things are working you can specify the maximum number of sequences to download. This is only to be used for testing and set to NULL by default.

Step 2 Run BUSCO

Once you have downloaded all the sequence data, you are ready to run BUSCO.

```
RunBusco(fasta_dir = fd, toast_dir = td, path_to_run_busco.py = bs, path_to_orthoDB = od, threads = cpu)
```

This function uses the paths you set up earlier to run BUSCO with a specified number of cores. Note that this step may take some time depending on the number of sequences and taxa you are searching.

Step 3 & 4 Parse and Extract Results

BUSCO generates a lot of results, so next you will parse out BUSCO IDs and extract the sequences of interest.

```
parsed_busco_results <- ParseBuscoResults(busco_dir = bd)

write.table(parsed_busco_results, file = paste0(td, "/parsed_busco_results.tsv"), sep = "\t", row.names = FALSE)

ExtractBuscoSeqs(busco_table = parsed_busco_results, fasta_dir = fd, extract_dir = ed) #parsed_busco_results from previous step
```

These lines go through the BUSCO directory, parse the IDs to generate a table that is both stored locally for your records using write.table and used to extract all orthologs.

Step 5 Align

All that is left to do is to align as follows

```
MafftOrientAlign(extract_dir = ed, mafft_dir = md, threads = cpu)
```

This will now generate alignments of each ortholog! To assemble and screen missing data head to section 4, or check the next section to see how local data could be added to this process.

Section 02

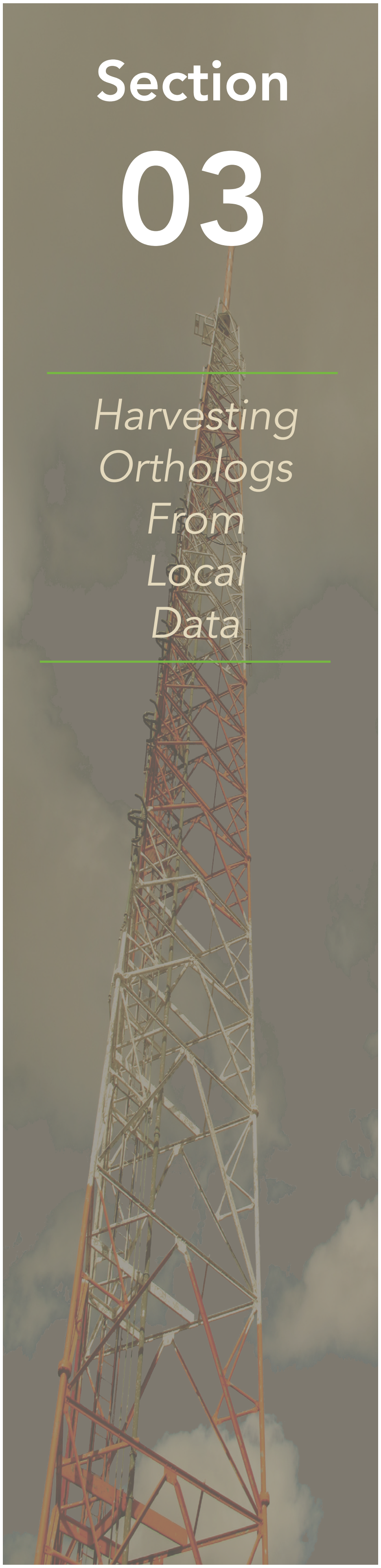

Harvesting Orthologs from local FASTA files

TOAST gives you the option to use BUSCO to either assemble new alignment from online data (previous section) or to use a combination of local and public files.

This means you can use TOAST to assemble orthologs from any local set of fasta files in a directory as covered in the previous section.

Local and/or Public Data

Should you already have a target set of FASTA files from downloading as above or may have generated novel data (assembled transcriptomes, genomes, etc). It is of course possible to use TOAST to harvest BUSCO orthologs locally and either skip the download step or add public data to your own!

To do this all you have to do is

Place your FASTA file/files into the
specified FASTA directory
in the prior section

Go through the code in section 2

That's it! Use any combination of local and public data to extract orthologs.

If you do not wish to download data or have already downloaded, simply omit 'Step 1 Download Data' on the preceding page.

Important note:
Remember to first set all of your directories and also your number of cores for BUSCO.

At this point you should now be able to use BUSCO to harvest orthologs from any FASTA files, provided you are using Unix. We encourage integration of public and novel data however caution that there can be high levels of missing data.

In the next section we will cover how to visualize missing data patterns and use this information to assemble custom concatenated alignments that meet user defined acceptable levels of data representation for a given problem.

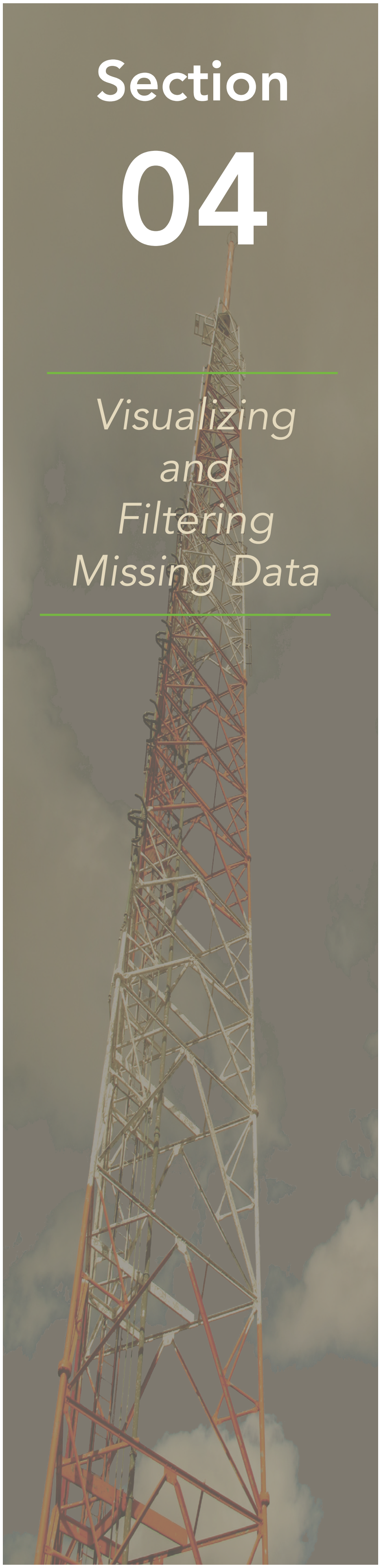

Missing Data

Missing data is a common feature of large sequence datasets. Everything from changes in coverage depth, DNA/RNA quality, probe design, assembly method, etc can result in uneven coverage of loci between species. TOAST provides a suite of functions to both explore missing data and reassemble alignments based on user determined thresholds of acceptable representation.

You will use the missing_data.tsv file distributed with TOAST in the examples in this section.

Note that you can use the following code to generate a .csv or .tsv file of missing data patterns from **ANY** set of FASTA files in a directory with the following function

```
setwd(td)
missing<-MissingDataTable(aligned_dir = ad)
write.csv(missing, file="missing_data.csv")
```

In fact, **ANY** delimited file of data presence/absence can be used with these visualization functions (just needs data and NA for no data). This includes phenotypic trait data, behavioural observations, etc!
Now you have no excuse to not look at missing data patterns anymore !

Visualizing Missing Data Patterns

Part of the orthology assembly steps in both sections 2 and 3 generates a .tsv file entitled "missing_data.tsv" that is distributed with this software. You will use this file throughout this section.
If not in memory, please be sure to read it into memory as below

```
tsv<-read.delim("~/missing_data.tsv", header=TRUE)
```

With this file you can begin to explore coarse missing data patterns. Let's start with a snapshot of which taxa have more than 1000 out of 6000 loci in the cetacean dataset as follows

```
VisualizeCoverage(tsv, 1000)
```

This will produce a two panel graphic. The top panel is a circlepack plot showing the number of missing loci per species that meet the threshold of having at least 1000 loci. The bottom panel depicts the number of loci captured versus the number missing for each species that did not meet the threshold criterion.

In this case, the taxa not meeting the threshold were very similar in having little sequence coverage. In contrast, *Tursiops* stands out as having the most missing loci among the taxa that did meet this criterion. Note that the lack of yellow in panel B reflects the lack of sequence data present in these species.

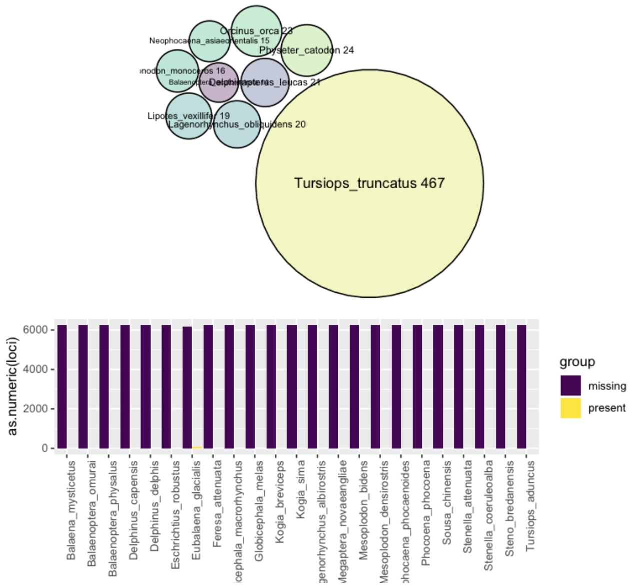

You may also be interested in simply knowing which taxa have complete coverage of all orthologs.

```
CompleteCoverage(tsv)
```

```
[1] "Balaenoptera_borealis"   "Balaenoptera_brydei"     "Cephalorhynchus_hectori" "Eubalaena_australis"     "Grampus_griseus"
[6] "Lagenorhynchus_acutus"   "Phocoenoides_dalli"      "Pseudorca_crassidens"    "Ziphius_cavirostris"
```

This provides a list of species with complete coverage.

As you assemble genome-scale coverage of the Tree of Life, it is clear that sequence data is unevenly distributed between groups of organisms. TOAST offers the ability to explore missing data interactively based on a user specified taxonomic framework. For each species, you may supply as many taxonomic/phylocode/etc levels as you like to explore the hierachical structure of missing data.

The guide file is formatted as follows

level1	level2	leaves
Mysticeti	Baleana	Balaena_mysticetus
Mysticeti	Baleanopteridae	Balaenoptera_acutorostrata
Mysticeti	Baleanopteridae	Balaenoptera_borealis
Mysticeti	Baleanopteridae	Balaenoptera_brydei
Mysticeti	Baleanopteridae	Balaenoptera_omurai
Mysticeti	Baleanopteridae	Balaenoptera_physalus
Odontoceti	Delphinidae	Cephalorhynchus_hectori
Odontoceti	Monodontidae	Delphinapterus_leucas
Odontoceti	Delphinidae	Delphinus_capensis
Odontoceti	Delphinidae	Delphinus_delphis
Mysticeti	Eschrichtiidae	Eschrichtius_robustus

Section 04

Leaves are your sampled species and there is no limit to the number of levels you can add. Simply add more columns if you wish for increased resolution. Please note that you need a column entitled “leaves” which follow the phylogenetic convention of designating your tip taxa as "leaves". You can save this in any format you wish to read into R (.csv, .tsv, etc). In our case, you will use a .csv file that is distributed with this software.

Additionally, you must also fill in all levels. For the purpose of this tutorial you will look at the difference between baleen and toothed whales and higher level subclades such as dolphins using your web browser to interactively explore missing data circlepack plots.

```
taxonomy<-read.csv("/toast/sample_data/cetacean_taxonomy.csv", header=TRUE)
#note, please change the path to reflect the location on your HD
VisualizeTaxonomyInteractive(tsv, taxonomy, 0)
```

As above, the missing data file along with your threshold of mininum number of loci is used to generate these plots. You are going to begin by setting our threshold to zero, to see how missing data is distributed overall. The above code should have opened an interactive plot in your browser like this one

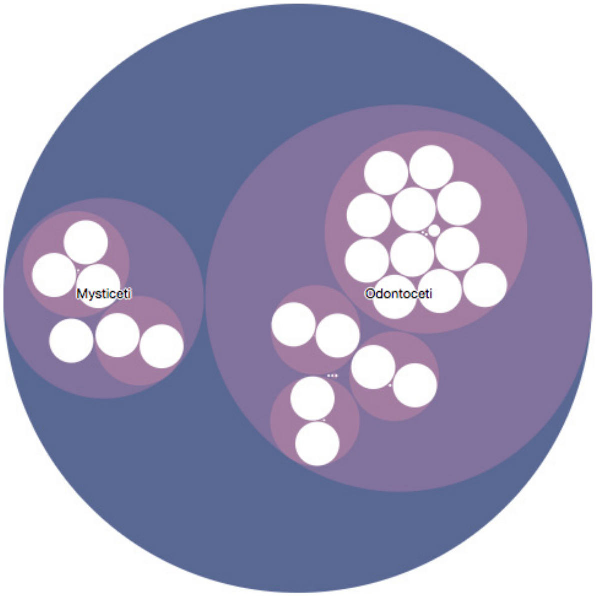

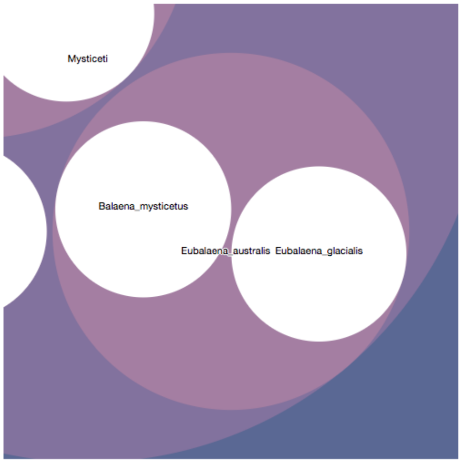

The size of the circles here indicate higher levels of missing data per leaf taxon. At a glance you can see that a big chunk of missing coverage is within a specific sphere of toothed whales (Odontoceti).

Clicking around this plot you can zoom in and out of these spheres to get more detail.

Section 04

To give an example with a threshold, let's again look at taxa with more than 1000 loci sampled.

```
VisualizeTaxonomyInteractive(tsv, taxonomy, 1000)
```

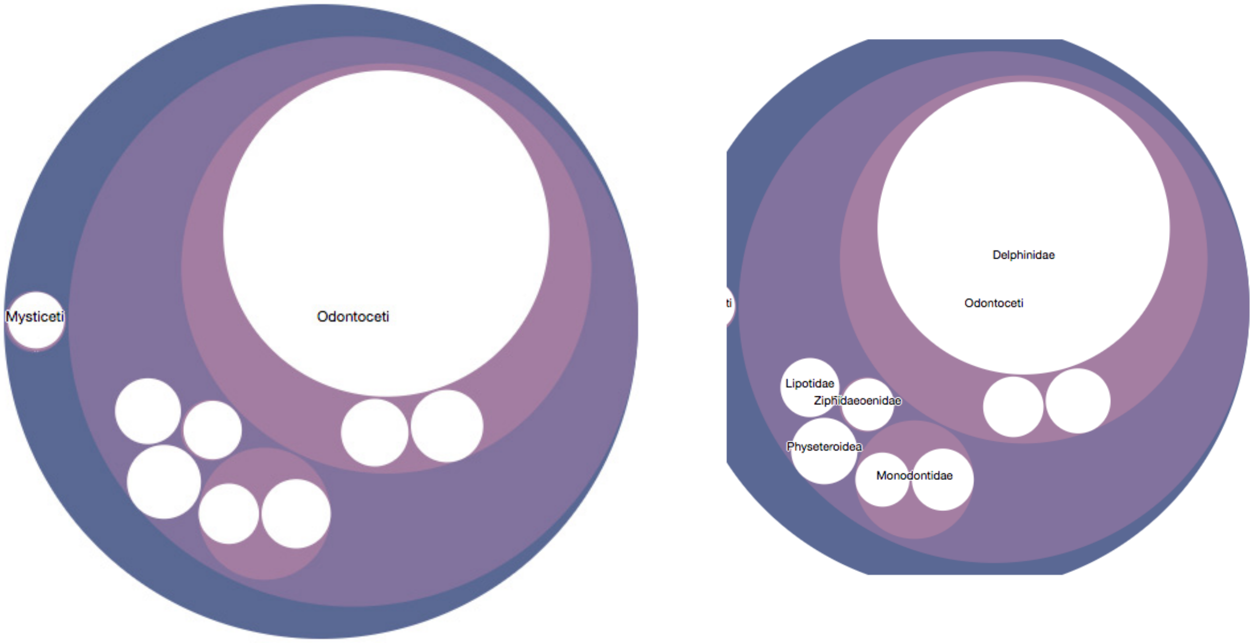

Again, there is more missing data in Odontoceti and clicking within the plot reveals that the species with the most missing data are within dolphins.

You may be interested in exploring how the setting of a missing data threshold changes the distribution of missing data pre and post filtration.

For example, merely saying you would like to have at least 1000 loci represented per species may mask the fact that for taxa meeting this threshold, each has substantially more.

Alternatively, a user threshold may leave a single taxon with exceedingly higher levels of missing data than others.

To visualize the impact of thresholding on missing data do the following

Section 04

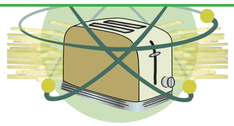

```
VisualizeThreshold(tsv, 1000)
```

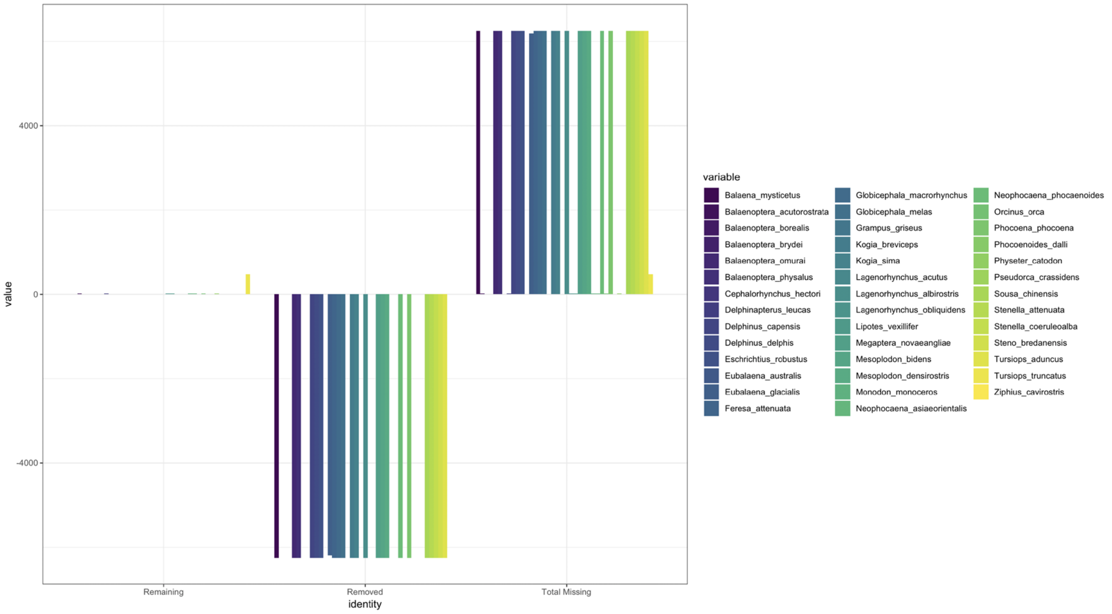

The above plots shows your total remaining missing data (left), how much missing data was removed (right), and how much missing data was in the originally present in your data (right). In each case the bars are color coded by taxa.

In addition to thinking about missing data between taxa, it may also be of interest to look between loci and taxa at different hierarchical levels.

This could reveal clade biases in missing data representation and also be of interest for the design of probe sets or sequence capture efforts given a small pilot dataset.

TOAST currently offers two visualizations that facilitate taxon specific visualization of missing values using the taxonomy file from earlier examples in this section. We will begin by looking at a barplot of missing data.

Note that this uses parallel processing so if you have not done so, define the number of processors to use as follows, changing the number of cores to what you have available on your machine.

```
library(doParallel)
registerDoParallel(cores=8)
```

```
MissingStreambar(tsv, taxonomy, "level1", 0, type="bar")
```

This code will generate a traditional stacked barplot of missing data across all loci by hierarchical level (in this case odontocetes versus mysticetes).

The Y axis is the frequency of missing data for each locus, color coded by hierarchy. The X axis is each locus.

We set the threshold to
zero here just to visualize raw missing data patterns. In this case, missing data is fairly evenly distributed across loci.

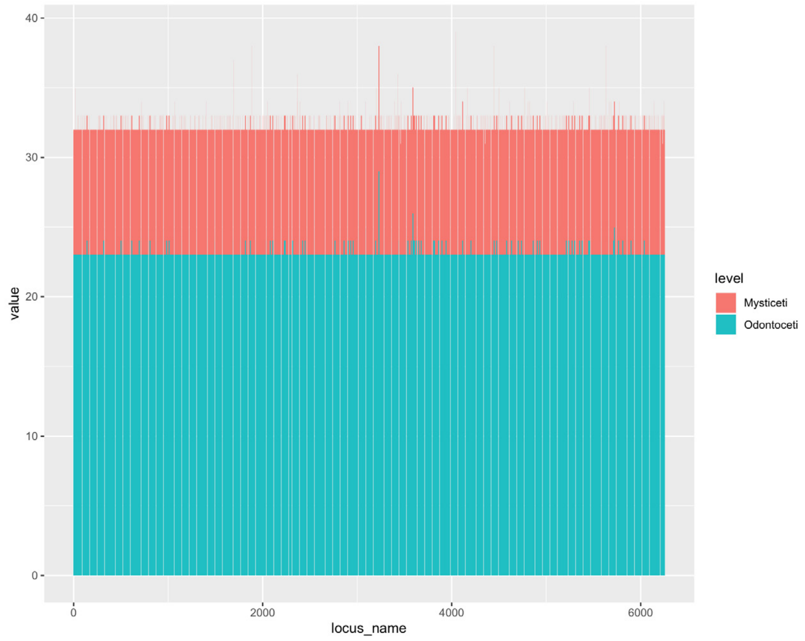

An additional spin on this plot is that for experimental design, you may wonder what sorts of missing data patterns could I expect given a probe set or other sequence capture method. One way to visualize this is to make use of streamgraphs which interpolate data values across a matrix.

If you have pilot data and are considering the potential for sequence capture (or really any data capture), you can generate an interactive streamgraph to see what sorts of missing data patterns you may expect should you continue to aggregate data using the same techniques as the pilot.

Prior to looking at this graph please do note that this assumes your pilot data is characteristic of dataset as a whole, so do use common sense and your own judgement in your experimental design choices as this is merely a prediction.

```
MissingStreambar(tsv, taxonomy, "level1", 0, type="stream")
```

The above code will open up an interactive plot in your web browser.

Section 04

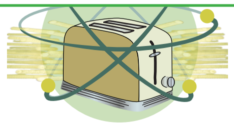

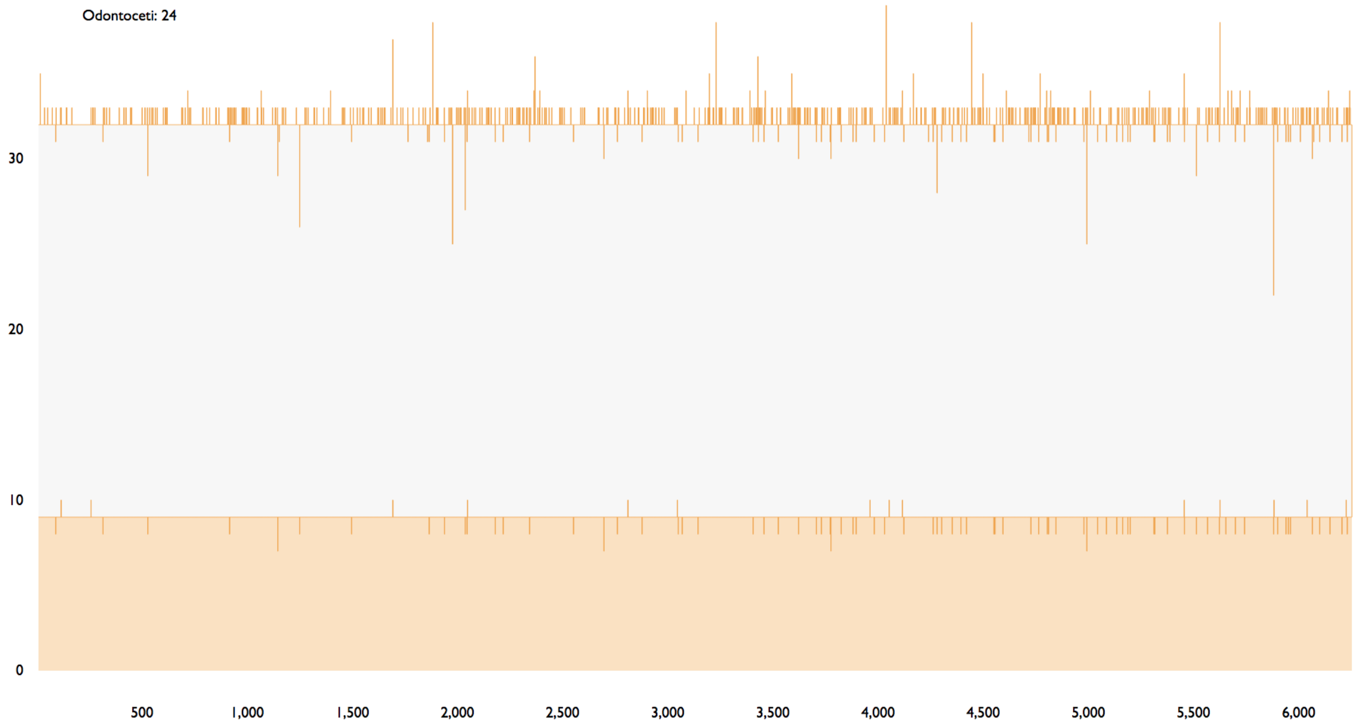

Now that you have looked at missing data patterns, you may want to reassemble your concatenated alignments. TOAST has a series of tools for doing just that!

Assembly of Sequence Datasets

TOAST has several concatenation functions.

The most basic will assemble a concatenated alignment of aligned fasta files into the relaxed phylip format used by IQtree, along with a nexus file of partitions that can also be read directly into IQtree for model/partition selection. You can use this function with ANY set of aligned fasta files in a directory. "missing" below refers to the output of the function: missing<-MissingDataTable(aligned_dir = ad).

```
SuperAlign(aligned_dir=ad, missing_df = missing)
PartitionTable(aligned_dir=ad, missing_df = missing)
```

Additionally TOAST will take your missing data threshold to remove taxa that do not meet the criterion for retention, realign all loci, concatenate the filtered dataset into the relaxed phylip format used

by IQtree, and generate a nexus file of partitions that can also be read directly into IQtree for model/partition selection. Note that TOAST does not come with a test data set for this out of the box, so you will either need to run TOAST on the example data or supply your own set of aligned fasta files. If you have this you can use the code below!

```
ThresholdDataTable(missing, threshold=1000)->threshold_df
ThresholdExtract(aligned_dir=ad, missing_df=threshold_df, threshold_fasta_folder="path/to/store/fastas/threshold100")
```

For the above the directories should be whatever you specified earlier on. Again we recommend the folder architecture at the start of this guide. At this point you should be ready to harvest and explore data. Remember to check back for updates as we add functionality and please drop a line if you have features you would like to see added.

Advanced Visualizations

This section is for users who wish to go further and more readily explore their data and have some proficiency in R. For users interested in how missing data patterns correspond to biological processes, TOAST can access GOterms assigned by BUSCO and generalize these into GOslim terms. This facilitates plotting missing data patterns binned by GO terms. Please note that these visualizations require you to decide what elements to emphasize and therefore are not functions. To begin, the ToastGoSlim function looks into the orthodb (e.g., Laurasiatheria database used above) to define user selected Goslim terms within an obo database.

The obo database can be accessed here:

http://geneontology.org/docs/download-ontology/#go_obo_and_owl

and requires downloading. These are updated frequently and we recommend keeping up with the latest version!

Once downloaded uncompress the orthogroup.info file within the info folder before proceeding.

Once the database has been downloaded AND EXTRACTED use the TOASTGoSlim function as follows:

```
BP <- ToastGoSlim(orthogroup_info = ortho, obo = go_oro, perspective = "BP")
#converts BUSCO_IDs into a dataframe of Biological Process GoSlim Terms
```

Section 05

If you wish to make UpSetR style plots you can first install the package

```
# check out their project here: https://github.com/hms-dbmi/UpSetR
if ("UpSetR" %in% rownames(installed.packages()) == FALSE) {
   install.packages("UpSetR")
}
```

UpsetR plots offer tremendous flexibility. As such we choose not to black box this tool and instead offer a walkthrough here that can be expanded upon for individual use.

Call the library and run these steps to prepare the BP object for the UpSetR function and plot. This mirrors the upsetR tutorial (address here) so we do not explain the lines in detail, but offer comments to supplement the existing tutorial.

```
library(UpSetR)
#Create intersection plot
#format BP data set to plot using the upset() function
rownames(BP)[21] <- "carb. derivative metabolic" #original name too long
to_plot <- t(BP) #transpose the data
to_plot[to_plot > 0] <- 1 #remove comparisons with no overlap
to_plot <- data.frame(to_plot) # convert to dataframe
to_plot <- cbind(rownames(to_plot), to_plot) #set rownames to be the first row, re-
move rownames later
colnames(to_plot)[1] <- "Identifier"
rownames(to_plot) <- NULL #remove rownames

#format BP data set to plot
to_plot[,2:ncol(to_plot)] <- sapply(to_plot[2:ncol(to_plot)], as.integer)

#create intersection plot
upset(to_plot, order.by = "freq", sets = colnames(to_plot)[2:ncol(to_plot)])
```

The above code will produce the following graphic (next page), visualizing overlapping GoSlim terms and their frequency in the data.

You can compare the upsetr plot to a classic occupancy matrix. This process begins by making a binary matrix of presence and absence values using the busco directory defined earlier in this manual. From there run and customize the following code depending on your goals. Since we don't know what every user wishes to plot, we offer the following lines and comments for customization.

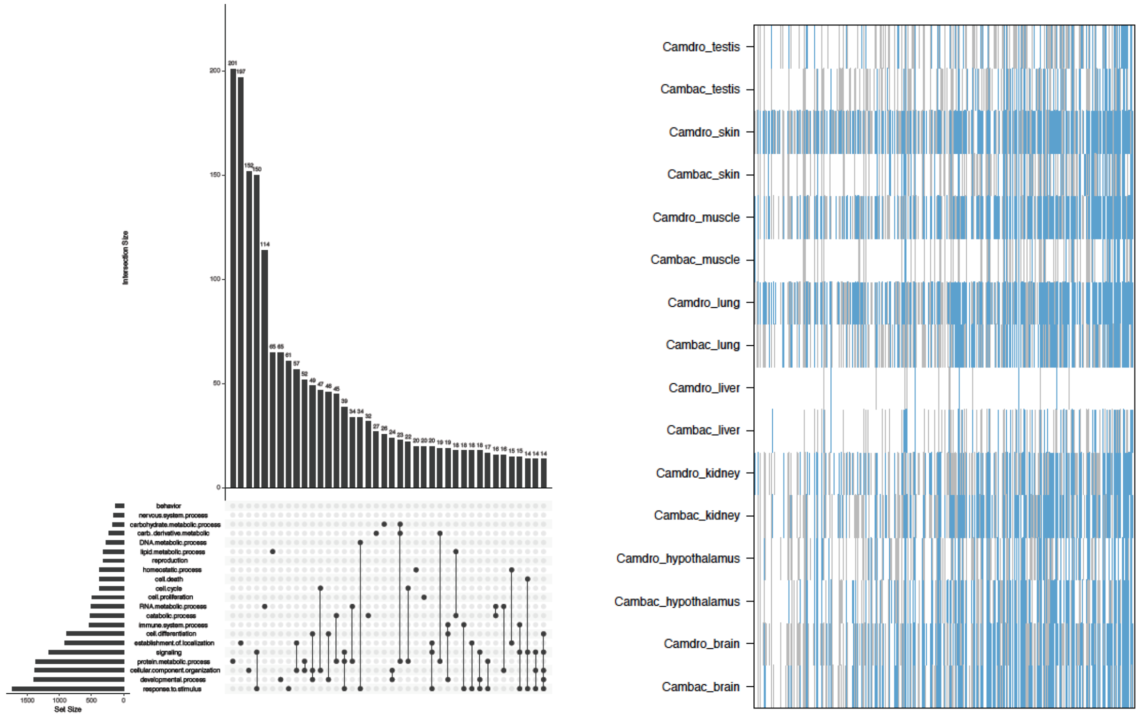

UpsetR plot (left) and occupancy matrix (right) depicting overlapping GOslim terms and missing data patterns.

```
### Create an occupancy matrix plot to display which Busco Ids were found in each
sample

VPBR <- VisualizePBR(busco_dir = bd) #creates a data.frame, similar to the function
ParseBuscoResults()

dim(VPBR) #check out the dimensions

vpbr <- as.matrix(VPBR[,2:length(colnames(VPBR))]) #convert VPBR data.frame to vpbr
matrix
vpbr <- gsub("Fragmented", 0.5, vpbr)            # format the data frame into a matrix
and replace Fragmented,
vpbr <- gsub("Complete", 1.0, vpbr)            # Complete, Duplicated and NA (ie
missing) with numeric values
vpbr <- gsub("Duplicated", 1.0, vpbr)
vpbr[is.na(vpbr)] <- 0

class(vpbr) <- "numeric" #image function below needs numeric or logical values to
plot

vpbr <- vpbr[,c("busco_Cambac_brain.fasta", "busco_Camdro_brain.fasta",
              "busco_Cambac_hypothalamus.fasta", "busco_Camdro_hypothala-
mus.fasta",
              "busco_Cambac_kidney.fasta", "busco_Camdro_kidney.fasta",
              "busco_Cambac_liver.fasta", "busco_Camdro_liver.fasta",
              "busco_Cambac_lung.fasta", "busco_Camdro_lung.fasta",
```

```
                    "busco_Cambac_muscle.fasta", "busco_Camdro_muscle.fasta",
                    "busco_Cambac_skin.fasta", "busco_Camdro_skin.fasta",
                    "busco_Cambac_testis.fasta", "busco_Camdro_testis.fasta")]

# Create y axis labels
yLabels <- gsub("busco_", "", colnames(vpbr))#remove the leading busco_ and trail-
ing .fasta
yLabels <- gsub(".fasta", "", yLabels)

ColorRamp <- gray.colors(3, start = 1, end = 0, gamma = 2.2, rev = FALSE) #white =
absent, gray = fragmented, black = duplicated/complete
Colors <- c("#FFFFFF", "#BABABA", "#57A0D3")
# Set layout
par(mar = c(5,15,2.5,1), font = 2) #mar=C(bot, left, top, righ 45-t)

# Make the plot
image(1:nrow(vpbr), 1:ncol(vpbr), vpbr,
     col=Colors, xlab="", ylab="",
     axes=FALSE, main= NA)

# Annotate the y axis
box()
axis(side = 2, at=seq(1,length(yLabels),1), labels=yLabels, las= 1,
    cex.axis=1)
```

If you are interested in visualizing the percent of each sample found within a GOterm category, this can be done using the following lines of code. Again this highly customizable using the following lines of code and comments as a guide.

```
# create color palette:
library(RColorBrewer)
coul <- brewer.pal(8, "Pastel1")
coul <- c(coul, brewer.pal(8, "Pastel2"))

data_percentage <- as.matrix(sorted_pie_df/rowSums(sorted_pie_df)*100)

class(data_percentage)

# Make a stacked barplot--> it will be in %!
end_point = 0.5 + nrow(sorted_pie_df) + nrow(sorted_pie_df)-1
par(mar = c(15,5,1,15))
barplot(t(data_percentage), col=coul , border="white", space = 1.0, xlab = "", ylab =
"Percent",
      main = "Percent Sample within GoTerm Category",
      axisnames = F)
```

```
text(seq(1.5,end_point,by=2), par("usr")[3]-0.25,
    srt = 60, adj= 1, xpd = TRUE,
    labels = paste(rownames(sorted_pie_df)), cex=0.9)

legend(x = 45, y = 100, legend = rev(colnames(sorted_pie_df)), fill = rev(coul), xpd =
1)
```

This produces the following plot:

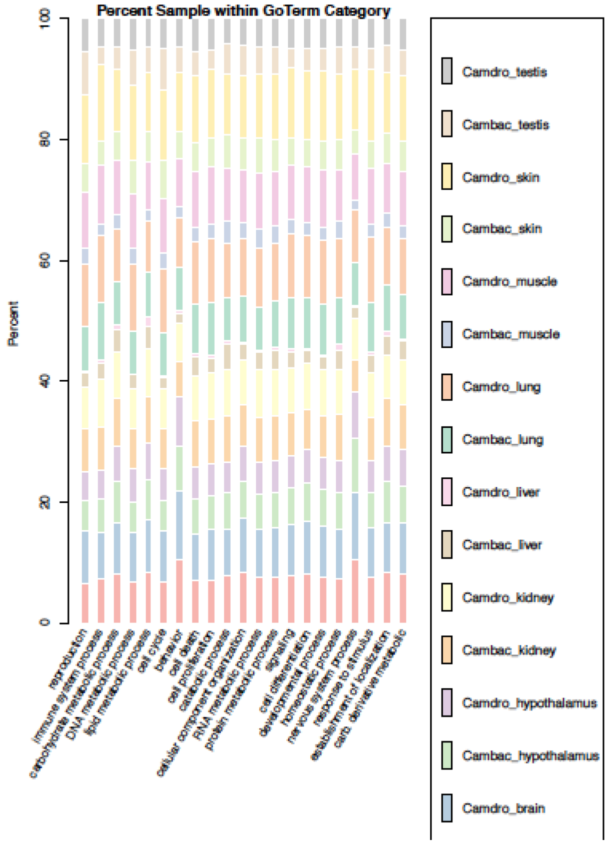

That's it for now, but keep checking for software and manual updates as TOAST gets further developed!

Acknowledgements

This research was supported, in part, by grants from the National Science Foundation (IOS-1755242 to AD and IOS-1755330 to JAY). We thank J. Vasquez for inspiration and E. Ferraro, K. Carlson, and E. Parker for comments on earlier versions of this manuscript as well as help with software documentation and tutorials. We would like to additionally thank participants of the 2019 Physalia phylogenetic comparative methods course for beta-testing an early version of this software.
